# Correction: Encapsulation of a photosensitizer into cell membrane capsules for photodynamic therapy

**DOI:** 10.1039/d4ra90097a

**Published:** 2024-09-12

**Authors:** Lijie Han, Ying Chen, Jie Niu, Lihua Peng, Zhengwei Mao, Changyou Gao

**Affiliations:** a Department of Polymer Science and Engineering, MOE Key Laboratory of Macromolecular Synthesis and Functionalization, Zhejiang University Hangzhou 310027 China zwmao@zju.edu.cn +86-571-87951108; b Institute of Pharmaceutics, College of Pharmaceutical Sciences, Zhejiang University Hangzhou P. R. China

## Abstract

Correction for ‘Encapsulation of a photosensitizer into cell membrane capsules for photodynamic therapy’ by Lijie Han *et al.*, *RSC Adv.*, 2016, **6**, 37212–37220, https://doi.org/10.1039/C6RA07480D.

The authors regret that an incorrect version of Fig. 9c was included in the original article. The correct version of Fig. 9 is presented here. An independent expert has viewed the corrected images and has concluded that they are consistent with the discussions and conclusions presented.

**Fig. 9:**
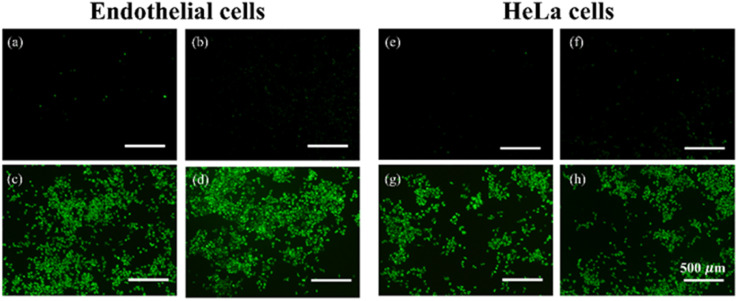
Fluorescence microscopy images of (a–d) the endothelial cells and (e–h) the HeLa cells after incubation in a medium containing 10 μM DCFH-DA and (a and e) CMCs, (b and f) CMCs + 10 J cm^−2^ irradiation, (c and g) 33.3 μM free MB + 10 J cm^−2^ irradiation, and (d and h) CMCs@MB containing 33.3 μM MB + 10 J cm^−2^ irradiation, respectively.

The Royal Society of Chemistry apologises for these errors and any consequent inconvenience to authors and readers.

